# Treating Acute *EX*acerbations of *C*OPD with Chinese Herb*AL* Med*I*cine to aid Anti*B*iotic *U*se *R*eduction (EXCALIBUR): study protocol of a randomised double-blind, placebo-controlled feasibility trial

**DOI:** 10.1186/s40814-022-01224-8

**Published:** 2022-12-19

**Authors:** Xiao-Yang Hu, Tom Oliver, Merlin Willcox, Catherine Simpson, Kerensa Thorne, Jeanne Trill, Nick Francis, Beth Stuart, Michael Thomas, Paul Little, Jian-Ping Liu, Gareth Griffiths, Michael Moore

**Affiliations:** 1 Primary Care, Population Science and Medical Education, Faculty of Medicine, University of Southampton, Southampton, UK; 2 Southampton Clinical Trial Unit, University of Southampton, Southampton, UK; 3 Pragmatic Trial Unit, Wolfson Institute of Population Health, Queen Mary University of London, London, UK; 4 Centre for Evidence-Based Chinese Medicine, Beijing University of Chinese Medicine, Beijing, China

**Keywords:** Herbal medicine, Exacerbation, Chronic obstructive pulmonary disease, Randomised controlled trial, Feasibility, Qualitative

## Abstract

**Background:**

Acute exacerbations of chronic obstructive pulmonary disease (AECOPD) are a major reason for consultations in primary care, hospital admissions, deterioration in function, and mortality. Despite the majority of exacerbations not being caused by bacteria, as many as 70% of patients who present in UK primary care with AECOPD are prescribed antibiotics as part of standard care. However, finding effective non-antibiotic treatments for COPD exacerbations is a priority to reduce antibiotic use. The Chinese herbal medicine Shufeng Jiedu® (SFJD) has the potential to reduce treatment failure and duration of hospital stay. This study aims to determine the feasibility of conducting a fully powered randomised, double blind, placebo-controlled clinical trial on SFJD for AECOPD in UK primary care.

**Methods:**

This study is a phase III, two-arm individually double blind, randomised, placebo-controlled feasibility trial with nested qualitative study, coordinated by the Southampton Clinical Trial Unit (SCTU). Patients aged ≥ 40 years, with a current AECOPD, presenting with increased sputum purulence/volume, or breathlessness, and for whom the GP is considering use of antibiotics, will be eligible to participate. We aim to recruit seven eligible participants per month and randomise them to receive either the patent Chinese herbal medicine SFJD capsules or placebo for 14 consecutive days and to follow-up for 12 weeks. The primary outcomes include the feasibility of recruitment, study retention, and the percentage of diary completion.

**Discussion:**

If this trial demonstrates the feasibility of recruitment, delivery, and follow-up, we will seek funding for a fully powered placebo-controlled trial of SFJD for the treatment of AECOPD in primary care.

**Trial registration:**

This trial is registered via ISRCTN on 1 July 2021, identifier: ISRCTN26614726.

**Supplementary Information:**

The online version contains supplementary material available at 10.1186/s40814-022-01224-8.

## Background

Chronic obstructive pulmonary disease (COPD) is a common, preventable, and treatable condition that is characterised by persistent respiratory symptoms and airflow limitation [1]. The point prevalence of COPD was estimated to be 3.92% (95% CI 3.52–4.32%) worldwide in 2017 [2]. In the UK, there are an estimated 3 million people with COPD [3], with approximately 1.17 million people diagnosed, representing 1.9% of the population [4].

COPD is a leading cause of morbidity and mortality, associated with significant economic burden. Due to its nature as a chronic disease, patients typically have an impaired quality of life and suffer from disability and impaired motility. As the third leading cause of death worldwide [5], COPD accounts for 5.72% of all-cause deaths with an estimated disability-adjusted life years (DALYs) rate at 1068/100,000 [2]. In the UK, COPD is estimated to result in around 1.4 million GP consultations and 130,000 emergency hospital admissions, with a direct cost to the NHS of £810–£930 million annually [6]. Current global projections indicate that COPD is set to increase in line with ageing populations. It is predicted to increase from causing 3 million deaths annually in 2010 [7] to 5.4 million deaths annually by 2060 [8].

Acute exacerbations of COPD (AECOPD) are acute worsening of respiratory symptoms that require additional therapy [1]. They are major reasons for deterioration in function and mortality, accounting for a large proportion of healthcare costs. Further economic impact is driven by loss of working days due to illness. Standard care of acute exacerbations includes antibiotics, and 70% of patients presenting with AECOPD in UK primary care are currently in receipt of antibiotics [9]. The most common causes of the exacerbations are bacterial infections, viral infections, and environmental triggers, with 49.6% (95% CI: 44.2–55.0) being triggered by bacterial infection [10] and 37.4% (95% CI: 35.9–38.8) by viral infection [11]. Antibiotics are sometimes used inappropriately in AECOPD and could be potentially reduced [12]. Patients with COPD are particularly at risk of antibiotic-resistant infections, as they may receive multiple courses and excessive use of antibiotics [13, 14].

Strategies to better target antibiotics can safely reduce prescribing [9]. Antimicrobial treatment in patients with COPD can reduce the infecting load without entirely eradicating organisms in the airways, leading to an increased risk of resistant bacteria [15]. Finding effective non-antibiotic treatments for COPD exacerbations is a priority to reduce antibiotic use.

Traditional herbal medicines have the potential to improve symptoms of acute respiratory tract infections [16–19] and hence reduce both the symptom burden of these illnesses and unnecessary antibiotic prescribing both nationally and internationally. The Chinese herbal medicine Shufeng Jiedu® (SFJD) capsule is a patent formula consisting of eight Chinese herbs, all of which are used traditionally for treating respiratory infections (Table 1). SFJD is already on the market in China for treating respiratory conditions, with promising evidence showing its effects in various acute upper RTIs [20] and community-acquired pneumonia [21].

**Table 1:** Composition of SFJD

Functional roles^1^	Botanical species	Common names and plant part	Pin Yin^1^	Amount (g)	Cultivated from
Sovereign	*Fallopia japonica* [22]	Japanese Knotweed, *rhizome*	Hu Zhang	0.45	Lu’an, Anhui
Minister	*Forsythia suspensa*	Weeping Forsythia, *fruit*	Lian Qiao	0.36	Yuncheng, Shanxi
	*Isatis indigotica*	Indigo Woad, *root*	Ban Lan Gen	0.36	Tsitsihar, Heilongjiang
Assistant	*Bupleurum chinense* [23]	Chinese thoroughwax, *root*	Chai Hu	0.36	Weinan, Shaanxi
	*Patrinia scabiosaefolia*	Yellow Flowered Valerian, *herb*	Bai Jiang Cao	0.36	Enshi, Hubei
	*Verbena officinalis*	Vervain, *herb*	Ma Bian Cao	0.36	Xiangyang, Hubei
	*Phragmites communis*	Reed, *rhizome*	Lu Gen	0.27	Anguo, Hebei
Envoy	*Glycyrrhiza uralensis*	Liquorice, *root*	Gan Cao	0.18	Dingxi, Gansu

^1^The prescription of SFJD following the basic principle of Chinese herbal medicine formulation, sovereign, minister, assistant, and envoy [君臣佐使] principle, where sovereign herb plays a major role in providing treatment effects for the main syndrome/symptom; minister herbs assist the sovereign herb to strengthen treatment effects and to treat the concurrent syndrome/symptom; assistant herbs assist the sovereign and minister herbs to strengthen the therapeutic effects, treat the secondary syndrome/symptom, or reduce the side effects of sovereign and minister herbs that may have; and envoy herb deliver the other herbs to the right place in the body [24]

A systematic review of randomised controlled trials has shown that when added to usual care for AECOPD, SFJD reduces treatment failure from 20.1 to 8.3% (11 trials; 815 patients; relative risk 0.43, 95% confidence interval [CI] 0.30 to 0.62; low certainty) and duration of hospital stay (2 trials; 79 patients; mean difference − 4.35 days, 95% CI − 5.28 to − 3.43 days), compared with usual care alone in Chinese population [25]. A comprehensive synthesis of evidence regarding SFJD safety identified no serious adverse events from clinical trials or pharmacovigilance data [26]. Minor adverse events included nausea/vomiting, diarrhoea, unspecified gastrointestinal discomfort, dizziness, and rash; however, these were not statistically significantly different to control groups. No substantive safety concerns were identified for SFJD for clinical use, excluding pregnant or lactating women [26].

Based on these studies, we hypothesise that SFJD may improve symptoms in people with AECOPD and therefore reduce the necessity for antibiotics, the risk and duration of admission to hospital, and the risk of relapse.

This study aims to determine the feasibility of conducting a fully powered randomised controlled trial of SFJD in addition to usual care for AECOPD in UK primary care. Specific objectives are to test feasibility of trial recruitment process and retention, intervention management and procedures, and collecting outcome measures. We will also explore participants’ experiences of the trial procedures and their views of taking SFJD for their AECOPD.

## Methods

### Study design

**Fig. 1:**
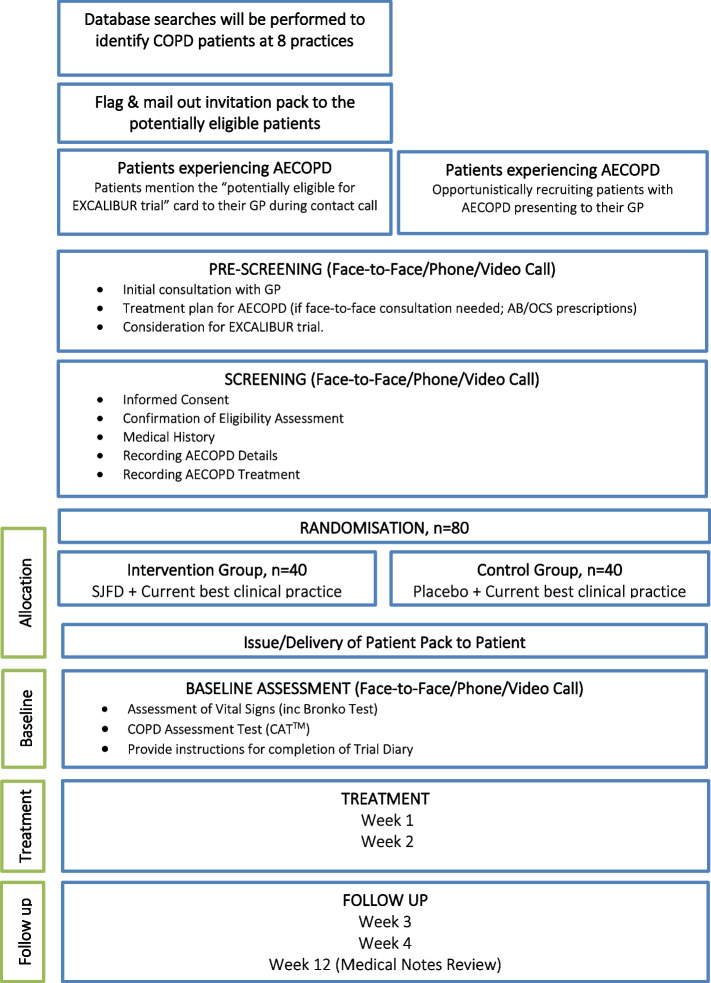
Trial schema

The EXCALIBUR study is a double-blind, randomised placebo-controlled feasibility trial (ISRCTN26614726) [27]. This trial protocol has received the favourable opinion of a Research Ethics Committee or Institutional Review Board (IRB) in the approved national participating countries (IRAS number 268737, Appendix 1; sponsor reference number 47948, Appendix 2; Ethical approval: REC reference 20/LO/0580, Appendix 3). A nested qualitative study will be undertaken with patients, including both those who agreed and declined to participate in EXCALIBUR (Fig. 1).

This protocol provides details for the study following the Standard Protocol Items for Clinical Trials (SPIRIT) 2013 statement [28] (Appendix 4). The final study results will be reported following the CONSORT Extension for Chinese Herbal Medicine Formulas [29], with the nested qualitative elements being reported using the COREQ checklist [30].

### Setting of the study

The study will be conducted in eight general practices in the Wessex region of the United Kingdom, coordinated by the Southampton Clinical Trial Unit (SCTU).

### Participants

The study selection criteria are outlined below. Patient eligibility to take part in the EXCALIBUR trial will be confirmed by a qualified GP or Nurse Prescriber. Evidence of eligibility criteria will be documented in the patient’s medical or research notes.

#### Inclusion criteria

Patients who fulfil all the following criteria will be recruited.

- Has a current acute exacerbation of COPD with at least one of the following:
  - Increased sputum purulence
  - Increased sputum volume
  - Increased breathlessness
- The current acute exacerbation has lasted for at least 24 hours and no longer than 21 days
- The responsible clinician is considering use of antibiotics for the acute exacerbation
- Diagnosis of COPD in clinical record
- Age 40 years or more
- Able to provide informed consent
- Able to provide the primary outcome data at 2 and 4 weeks

#### Exclusion criteria

- The responsible clinician feels urgent referral to hospital is necessary
- Severe illness, e.g. suspected pneumonia or pulmonary embolism or lung cancer; necessity for emergency admission to hospital
- Patient has a primary diagnosis of bronchiectasis, lung cancer or other active chronic respiratory disease
- Currently on or has previously had antibiotics or corticosteroids for this episode of AECOPD
- Patient is on a maintenance dose of antibiotics for treatment of COPD
- Known or suspected pregnancy
- Women of childbearing potential who are at risk of pregnancy and not using an effective form of contraception
- Currently breast-feeding
- Chronic kidney disease stage 4 or 5
- Severe liver disease
- Cannot read or understand the study materials
- Previously recruited into this ‘EXCALIBUR’ trial
- Previously recruited into another drug trial within the last 6 weeks

### Sample size

The target sample size for this trial is 80 patients (40 per arm), which means recruiting seven eligible participants per month. Patients will receive either SFJD or placebo capsules in 1:1 allocation ratio.

As this is a feasibility trial, no formal sample size calculation was carried out. However, using a 95% confidence interval approach and conservatively assuming a 50% participation rate (to give the worst-case scenario), it can be shown that this sample size allows us to predict the recruitment rate to within ± 13% [IBM SPSS Statistics for Macintosh, Version 25.0].

### Recruitment and consent

As clinical care has not yet returned to pre-pandemic patterns, both face-to-face and remote recruitment will be carried out.

Potential participants will be identified in several ways:

1. Participating practices will search their electronic records for patients on their COPD register and flag those who suffered one or more exacerbations in the last 5 years. These patients will then receive a mailed trial introduction pack to allow them to easily identify themselves when they contact the surgery.
2. Patients who fulfil this criterion at other practices in the participating practice’s Primary Care Network (PCN) may also be contacted and informed about the trial.
3. Participating practices may screen and recruit AECOPD patients who present opportunistically at their practice or at practices in their PCN.
4. Advertisement through trial posters in surgery waiting areas.
5. Practices will advertise the trial on platforms such as practice websites and social media accounts.

Potentially eligible patients will be sent an invitation letter by Docmail informing them about the study, a summary participant information sheet (PIS), and a patient card with ‘potentially eligible for EXCALIBUR Trial’. Participating clinicians will be asked to approach potentially eligible patients opportunistically in triage sessions. The GP will initially assess the patient and make a clinical decision on treatment as per standard care. Should the patient be deemed potentially eligible for the EXCALIBUR trial, they will be provided with a PIS and if the patient recruitment process is being conducted remotely a link to the trial consent website containing an electronic PIS. They will be allowed sufficient time to decide whether to participate and ask any questions they may have.

Informed consent will be taken face-to-face or through the trial consent website using a bespoke e-consent platform. The right of the patient to refuse to participate without giving reasons will be respected. All participants will be free to withdraw from the trial at any time without providing reasons and without prejudicing further treatment. Participants in the trial who are potentially interested in being interviewed will be asked for consent to share their contact details with the qualitative researcher. PIS and consent form are available in Appendix 5.

Following explicit consent from the participant to do so, participant details will be collected on the trial website for the purpose of conducting the trial. For randomised participants who are recruited remotely, the trial medication and participant diaries/questionnaires will be delivered to their home address via dedicated courier.

### Confidentiality

SCTU will preserve the confidentiality of participants taking part in the trial. The investigator must ensure that participants’ anonymity will be maintained and that their identities are protected from unauthorised parties. On CRFs participants will not be identified by their names, but by an identification code. For the qualitative aspects of the study, transcripts will be anonymised before analysis.

### Randomisation

Eligible participants who have provided written or online informed consent will be individually randomised in a 1:1 allocation ratio to receive SFJD or placebo treatment. The randomisation sequence was generated using block randomisation with no stratification factors with Stata version 16.0 (StataCorp LLC) by a statistician [KT] at the SCTU. Randomisation codes were securely sent by the statistician [KT] to a labelling and packaging (design approved by the study sponsor) technician at Anhui Jiren Pharmaceutical Co., Ltd. China, who was not involved in the administration of the trial. Once a patient has been randomised, the recruiting site will inform the SCTU by scanning and emailing through the eligibility-randomisation form within 24 h of randomisation.

The treatment packs are sent to site in sets of four and each patient will receive the next available sequentially numbered patient pack at their site. This will determine their patient identifier number. The doctor or nurse allocating the patient pack and the patient will not know to which treatment arm they have been randomised. The patient pack will contain either SFJD or placebo capsules. Outcome assessors will also be blind to treatment allocation.

### Blinding

The participant, as well as the GP, research nurse, HCA, or RA allocating the patient pack will be blinded to which arm they have been randomised. The patient packs containing either SFJD capsules or placebo capsules are provided in identical packaging to ensure blinding.

An emergency unblinding service is not required for this trial due to the low risk of the Investigation Medicinal Product (IMP) treatments. This has been documented in the trial risk assessment. Any emergency clinical decisions required will not be affected or altered by knowledge of the treatment group allocated to the patient. If unblinding is required this can be done by the Trial Statisticians at the SCTU.

### Intervention

In addition to receiving the current best practice usual care as informed by the local guidelines, participants will receive either 4 × 520 mg capsules of SFJD (Z20090047, batch No. 3210501) or 4 × 520 mg capsules of placebo (batch No. 3210601) identical in appearance: hard capsule, dark brown to reddish-brown granules or powder as content; similar in odour: SFJD aromatic, placebo burnt and aromatic; and different in taste: SFJD bitter, placebo sweet. Both SFJD and placebo are to be taken three times a day, preferably after meals, for a period of 14 consecutive days. The SFJD capsules contain eight herbs (Table 1), with 0.52 g extract, 0.05 g corn dextrin, and 0.05 g silicon dioxide per capsule.

The placebo capsule excipients are corn dextrin (79.66%), caramel (4.62%), food additive lemon yellow (0.35%), compound colourant chocolate brown (0.05%), compound colourant gardenia yellow (0.19%), compound colourant Cocoa Brown (0.23%), naringin (9.62%), anhydrous citric acid (0.96%), menthol (0.96%), FA-10101 sauce flavour essence (2.88%), and MCK135C ginger powder base (0.48%).

A delayed antibiotic prescription strategy will be encouraged by the patients’ responsible clinician; they will be able to offer either immediate antibiotics, delayed antibiotics, or no antibiotics. If antibiotics are prescribed, the choice of antibiotic will be a clinical decision made by the responsible clinician with reference to local guidelines. Common first-choice oral antibiotics for AECOPD include amoxicillin, doxycycline, and clarithromycin [31]. The recruiting GPs will make a clinical decision whether to provide additional treatment, e.g. oral corticosteroids, based on their comprehensive assessment of the patient considering risks and benefits, as per local guidelines.

### Manufacturing process and quality control of the investigational medicinal product

Before the herbs enter the factory, professional and technical personnel check the appearance characteristics of the eight medicinal materials and identify them according to the appearance characteristics specified in CP 2015 to ensure that the herbs are correct. In addition, intermediate products have undergone impurities, identification such as HPLC, microbiology, assay, and description control testing at the company’s laboratory. All eight herbs included in SFJD are processed according to the Chinese pharmacopoeia 2015 Volume 1 (P52/P86/P164/P170/P205/P208/P280) or the Hunan Chinese Herbal Medicine Standard 2009 (P17 for Patrinia Scabiosaefolia). Flowcharts of the manufacturing process including cultivation, harvesting, drying, and cleaning the herbs are provided in Appendix 6.

A sample of each herb was retained and stored in the Central Laboratory Sample Retention Room at Anhui Jiren Pharmaceutical Co., Ltd. Samples are kept at a temperature between 10 and 30 °C and below 35–75% relative humidity. They are packed in sealed polyethylene bags. The retained sample volume of each batch of eight pharmaceutical plant materials is greater than 100 g.

Inspection report of finished product SFJD capsule suggested the product contains emodin (C_15_H_10_O_5_) over 3.0 mg, polydatin (C_20_H_22_O_8_) over 3.0mg, and forsythin (C_27_H_34_O_11_) over 2.0mg per capsule. The shape and properties, identification, content uniformity, and microbiology comply with the specifications according to the China National Medical Products Administration Standard YBZ05182019, 2020 version of Chinese Pharmacopoeia (Inspection Report No.: C-4-018, dated Jun 2021).

**Fig. 2:**
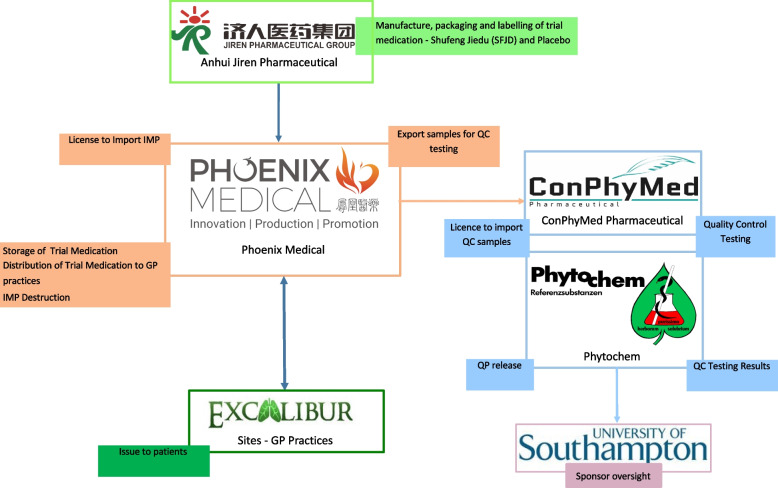
EXCALIBUR IMP flow chart

Quality assurance processes involving the manufacture, supply, and processing of the herbal medicines, certificates of analysis and stability testing of SFJD and placebo, and transmissible spongiform encephalopathy (TSE) testing and bovine spongiform encephalopathy (BSE) monitoring were performed in China. The trial investigational medicinal product (IMP) was then delivered from China to the UK (Fig. 2). Confirmation quality control testings were performed by ConPhyMed GmbH (Germany) and signed off by a qualified person according to the European Manufacturer’s Good Manufacturing Practice (GMP). IMP dossiers containing safety profile of the product were developed for SFJD capsule and its placebo.

### Trial discontinuation

In consenting to the trial, participants have consented to the trial intervention, follow-up, and data collection. Participants may be withdrawn from the trial procedures at any time, in the event of:

- Clinical decision, as judged by the principal investigator
- The development of toxicity, regardless of causality, which, in the investigator’s opinion, precludes further treatment under this protocol
- The patient withdraws consent
- Non-compliance with protocol

Full details of the reason for trial discontinuation will be recorded in the eCRF and medical record.

### Outcome measurements and data collection

As this is a feasibility study, only feasibility endpoints will be assessed:

1). Recruitment process and retention

- Eligibility: Proportion of patients on the COPD register who present with AECOPD
- Eligibility: Proportion of AECOPD-presenting patients eligible and ineligible (plus reasons) for the trial
- Recruitment/randomisation: Proportion of eligible patients recruited/randomised
- Recruitment: Rate of recruitment per month open in the UK primary care setting
- Retention: Across the duration of the trial

2). Intervention management and procedures

- Intervention compliance according to diary data and returned medication
- Average no. of capsules taken per day per patient
- Duration of treatment per patient
- Determine issues around safety and ADR reporting
- Effectiveness of blinding: Proportion of patients correctly guessing treatment/placebo allocation and reasons why

3). Completion of outcome measures

- Proportion of diary completion
- Proportion of patients returning trial diaries
- Proportion of patients who took antibiotics in each group
- Proportion of patients given immediate and delayed antibiotic prescriptions

Participants’ demographic data, their medical history, AECOPD symptoms, COPD Assessment Test (CAT)^TM^ symptom questionnaire, the EXAcerbations of Chronic Pulmonary Disease Tool - Patient-Reported Outcome (EXACT-PRO®) symptom questionnaire, and any adverse events during the trial process will be collected alongside a treatment diary, at various time points (Table 2).

**Table 2:** Schedule of observations and procedures

Observation/procedure		Timings of visit/contact						
	Person undertaking the specified event	Screening/registration Days 0–1	Treatment Week 1 Days 1–7	Treatment Week 2 Days 8–14	Follow-up Week 3 Days 15–21	Follow-up Week 4 Days 22–28	Follow-up Week 5 Day 35	End of study Week 12 Day 84
Informed consent	GP/nurse^1^/HCA^1^/RA^1^	**X**						
Eligibility evaluation	GP/nurse prescriber^1^	**X**						
Relevant medical history	GP/nurse/HCA	**X**						
Assess AECOPD symptoms	GP/nurse/HCA	**X**						
Prescribe antibiotics (as appropriate)	GP/nurse prescriber	**X**						
Issue trial medication/randomisation	GP/nurse/HCA/RA	**X**						
Issue treatment/symptom questionnaire diary	GP/nurse/HCA/RA	**X**						
Vital Signs	GP/nurse/HCA	**X**^**9**^						
Completion of CAT^TM^ symptom questionnaire^3^	GP/nurse/HCA/RA/patient^3^	**X**^**3,9**^		**X**^**3**^		**X**^**3**^		**X**^**3**^
Demographic data*	Patient		**X**					
Completion of EXACT-PRO® symptom questionnaire^2^	Patient		**X**	**X**	(**X**)^2^	(**X**)^2^		
Completion of treatment diary	Patient		**X**	**X**	**X**	**X**		
Phone call to patient (questionnaire assessment)^4^	SCTU		**X**^**4**^	**X**^**4**^		**X**^**4**^		**X**
Completion of diary/questionnaire by recall^5^	SCTU and patient						**X**	**X**
Adverse event (AE) notification^6^	SCTU and patient		**X**	**X**	**X**			
AE assessing^6^	GP		**X**	**X**	**X**			
AE recording/reporting^6^	GP/nurse/HCA		**X**	**X**	**X**			
Concomitant medication (only to be recorded in the event of an SAE and specified AEs)^7^	GP/nurse/HCA		**X**	**X**	**X**			
Serious adverse event (SAE) assessing	GP		**X**	**X**	**X**			
SAE reporting	GP/nurse/HCA		**X**	**X**	**X**			
Medical notes review	GP/nurse/HCA							**X**
Qualitative interview (refuse to participate in the trial)^8^	Qualitative researcher	(**X**)^8^						
Qualitative interview (participated in the trial)	Qualitative researcher						**X**^**10**^	

*NR* nurse researcher, *HCA* healthcare assistant, *RA* research assistant

*The relevant demographic and other data to be recorded by the patient on the first day of treatment are as follows: gender, employment, ethnicity, and smoking history

^1^In line with local GP surgery procedures with demonstrable and appropriate level of training. Specific duties delegated by the PI

^2^The EXACT-PRO® symptom questionnaire should be completed daily from day 1 of treatment until either the 14 day treatment is complete and resolution of their AECOPD symptoms have been maintained for 7 days or 28 days post-randomisation

^3^The CAT^TM^ symptom questionnaire should be completed by the patient with the site team staff on day 1 and by the patient in the patient diary on days 14, 28, and 84

^4^Patient phone calls to be completed on days 3, 14, and 28 to ensure the diary instructions provided by the research team at baseline are being followed and to answer any questions the patient has regarding diary completion. The SCTU team follow a trial script to ensure the requirements for collecting data for each endpoint are understood by the patient

^5^Only to be completed if the participant has not returned their participant diary

^6^Only AEs believed related to the trial medication will be recorded on the trial. Reporting and recording of all AEs related to the trial medication is carried out by GP/nurse

^7^Concomitant medication should only be recorded in the event of a serious adverse event or an adverse event related to the trial medication. When an SAE or trial medication-related AE occurs, all concomitant medication that the patient was taking at onset of the event should be recorded in the eCRF

^8^This interview may take place at any time between confirmation of a patient’s refusal to enter the trial and the end of the trial

^9^Vital signs and baseline CAT questionnaire may be completed on day 0 or 1, depending day of provision of patient pack to patient

^10^Qualitative interviews will take place at any time from day 35 onwards

NB: The participant is free to withdraw consent at any time without providing a reason. When withdrawn, the participant will continue to receive standard clinical care. Follow-up data will continue to be collected (unless the participant has specifically stated that they do not want this to happen).

All participant data for the main feasibility trial will be collected and uploaded to the Medidata RAVE® Electronic Data Capture database, which is hosted on servers based in the USA. However, the Medidata team will not have access to the trial data, and no data will be sent outside the UK. Baseline and medical notes review data will be collected and entered onto the database by research site staff, whilst information from the participants’ diaries and questionnaires will be transcribed and entered by the SCTU.

The expectation is for trial participants to complete the 28-day Trial Medication and Symptom diary and then for completion of the final CAT questionnaire and Notes Review to take place on day 84 post-randomisation. As the data collected from participants in the first 28 days are the most critical to the trial, and to maximise overall recruitment, patients will continue to be recruited until 28 days prior to the planned last patient last visit (LPLV) date. These participants will therefore have a truncated follow-up period: participants recruited between 84 and 56 days prior to LPLV will continue to receive their final CAT questionnaires, for completion on the LPLV date; and Notes Reviews will be performed on all participants, but with the period of review shortened to match the LPLV date. All necessary considerations will be made when including these patients in the trial analyses.

At the end of the trial after all queries have been resolved and the database frozen, the PI will confirm the data integrity by electronically signing all the eCRFs. Each PI will receive a copy of the original participant diaries for their site, as these are considered source documents, to allow the full PI oversight of the data. The eCRFs will be archived according to SCTU policy and a PDF copy including all clinical and Meta data returned to the PI for each participant. Data from the qualitative substudy will be transcribed and analysed separately.

### Statistical analysis

Study data will be entered onto SPSS (v.25) for data cleaning, coding, and analysis. The analysis of this feasibility trial will be mainly descriptive focusing on estimation rather than hypothesis testing. All baseline measures and outcomes will be summarised for each allocated group using the appropriate descriptive statistics. No formal comparison of groups will take place. A full statistical analysis plan will be developed prior to the final analysis of the trial.

### Trial oversight groups

The day-to-day management of the trial will be co-ordinated through the SCTU and oversight will be maintained by the trial management group and the trial steering committee. No independent data monitoring or data monitoring and ethics committees will be convened for this trial. These roles will be assumed by the trial steering committee.

### Patient and public involvement

The EXCALIBUR trial has benefitted from patient and public involvement (PPI). The trial management group (TMG) has two PPI members [JH, NG]; the trial steering committee has one PPI member [EC]; and a further two PPI members [DS, PH] were consulted alongside the TMG members when developing the protocol and trial materials, such as the participant diary. They have provided additional advice on the trial design, the protocol, and patient-facing study documentation. Potential barriers to participation and the outcome relevant to patients were discussed with them thoroughly, in consideration of the COVID pandemic situation. PPI will continue throughout the conduct of the trial.

We plan to utilise the skills of our PPI representatives to help interpret the qualitative data and to reflect on changes which may enhance recruitment and retention to the full trial if necessary. At the end of the study, it is important that the findings reach patient/public audiences and that the clinical audiences hear from the public voice; hence, we will include our PPI representatives in relevant presentations and/or articles to ensure maximum impact.

## Results

Data collection commenced in January 2022 and finished in September 2022. There was no interim analysis. The results are anticipated in late December 2022.

## Discussion

To the group’s knowledge, this is the first clinical trial on herbal remedies with multiple constituents in UK primary care. Although not being labelled as a Clinical Trial of an Investigational Medicinal Product (CTIMP) by the Medicines and Healthcare products Regulatory Agency (MHRA), as a feasibility trial, the team has gathered information required and formed the IMP dossier, placebo dossier, and a full safety dossier following the CTIMP requirements, as requested by the trial sponsor.

Adaptations that will be made due to the COVID-19 pandemic will be reported. Protocol amendments were submitted to REC and the sponsor for approval. If this trial demonstrates that recruitment and delivery are feasible, further funding will be applied for a fully powered placebo-controlled trial of SFJD for AECOPD in primary care. The results will be disseminated to patients and clinical teams through peer-reviewed journal publications and presented at international conferences, with the help of the PPI representatives.

Findings of this project will be shared with PPI representatives, healthcare professionals, and the public through meetings, conferences, and publications.

## Trial status

Participants’ recruitment started in January 2022 and completed by the end of July 2022. The last patient/last visit was at the end of August 2022.

### Trial sponsor

The trial sponsor is the University of Southampton. The contact person is Alison Knight. SCTU, the chief investigator [MM], and other appropriate organisations have been delegated specific duties by the sponsor, and this is documented in the trial task allocation matrix. These include but are not limited to: management of serious adverse events/reactions and onward reporting of SUSARs, management of deviations, and onward reporting of potential serious breaches.

The duties assigned to the trial sites (NHS Trusts or others taking part in this trial) are detailed in the non-commercial agreement.

There is no planned continued provision of the intervention after the research has finished. In the unlikely event that the participant is harmed as a result of taking part in the EXCALIBUR trial, there are no special compensation arrangements.

### Site and participant payments

The payments assigned to the trial sites (NHS Trusts or others taking part in this trial) are detailed in the study-specific site agreements. Agreed service support costs will be paid by the local CRN.

Patients entering the main trial will receive £10 as a thank you for their participation. Patients who take part in the qualitative interviews will receive £20 as a thank you.

## Supplementary Information

**Appendix 1.** IRAS number 268737.

**Appendix 2.** Sponsor reference number 47948.

**Appendix 3.** Ethical approval: REC reference 20/LO/0580.

**Appendix 4.** Standard Protocol Items for Clinical Trials (SPIRIT) 2013 statement.

**Appendix 5.** PIS and consent form.

**Appendix 6.** Flowcharts of the manufacturing process including cultivation, harvesting, drying and cleaning the herbs.

## Data Availability

In order to meet our ethical obligation to responsibly share data generated by interventional clinical trials, SCTU operate a transparent data sharing request process. As a minimum, anonymous data will be available for request from 3 months after publication of an article, to researchers who provide a completed data sharing request form that describes a methodologically sound proposal, for the purpose of the approved proposal, and if appropriate a signed data sharing agreement. Data will be shared once all parties have signed relevant data sharing documentation. Researchers interested in our data are asked to complete the Request for Data Sharing form (CTU/FORM/5219) [template located on the SCTU web site, www.southampton.ac.uk/ctu] to provide a brief research proposal on how they wish to use the data. It will include the objectives, what data are requested, timelines for use, intellectual property and publication rights, data release definition in the contract and participant informed consent, etc. If considered necessary, a data sharing agreement from sponsor may be required.
