# Supplementary material for: Treating Acute EXacerbations of COPD with Chinese HerbAL MedIcine to aid AntiBiotic Use Reduction (EXCALIBUR): study protocol of a randomised double-blind, placebo-controlled feasibility trial
Source: Pilot Feasibility Stud. 2022 Dec 19;8:262. doi: 10.1186/s40814-022-01224-8 (PMC9761047; doi:10.1186/s40814-022-01224-8)
Supplement: Supplementary file 2 — Appendix 2. Sponsor reference number 47948. [file 40814_2022_1224_MOESM2_ESM.pdf]

16 March 2020

Project title: EXCALIBUR - Treating Acute EXacerbation of COPD with Chinese HerbAL MedIcine to aid AntiBiotic Use Reduction

ERGO submission number: 47948

This letter is to confirm that the University of Southampton has agreed to act as Sponsor for the above research study under the terms of the UK Policy Framework for Health and Social Care Research (2017). We encourage you to become fully conversant with the terms of this Policy Framework (UKPF):

https://www.hra.nhs.uk/planning-and-improving-research/policies-standards-legislation/uk-policy-framework-health-social-care-research/

Sponsorship will remain in effect until the completion of the study and the ongoing responsibilities of the Chief Investigator have been met. Should the Chief Investigator fail to notify the Research Integrity and Governance Team of an amendment to the study, this may result in incorrect indemnity or sponsorship cover and may invalidate our agreement to sponsor.

If your study has been designated a Clinical Trial of an Investigational Medicinal Product, I would like to remind you of your responsibilities under the Medicines for Human Use Act regulations (2004/2006), The Human Medicines Regulations (2012) and EU Directive 2010/84/EU regarding pharmacovigilance. If your study has been designated a 'Clinical Investigation of a Medical Device' you also need to be aware of the regulations regarding conduct of this work.

Further guidance can be found:

http://www.mhra.gov.uk/

The University of Southampton fulfils the role of Sponsor in ensuring management, monitoring and reporting arrangements for research. As the Chief Investigator you are responsible for the daily management for this study, and you are required to provide regular reports on the progress of the study to the Research Integrity and Governance Team on this basis.

Please also familiarise yourself with the Terms and Conditions of Sponsorship attached, including reporting requirements of any Adverse Events to the Research Integrity and Governance Team and the hosting organisation.

If your project involves NHS patients or resources please send us a copy of your NHS REC and Trust approval letters when available. Please also be reminded that you may need a Research Passport to apply for an honorary research contract of employment from the hosting NHS Trust: https://intranet.soton.ac.uk/sites/researcherportal/Lists/Services1/testing.aspx?ID=607&RootFolder=%2A

Failure to comply with our Terms may invalidate your ethics approval and therefore the insurance agreement, affect funding and/or Sponsorship of your study; your study may need to be suspended and disciplinary proceedings may ensue.

Please do not hesitate to contact this office should you require any additional information or support. I would like to take this opportunity to wish you every success with your research.

Yours sincerely

Dr Alison Knight

Research Integrity and Governance Team

rgoinfo@soton.ac.uk

Tel No. 02380 598580

UNIVERSITY OF Southampton

Terms and Conditions of Sponsorship

Formal acceptance by the University of Southampton (UoS) to act as Sponsor for a research study as required by the UK Policy Framework for Health and Social Care Research 2017 (UKPF) and The Medicines for Human Use (Clinical Trials) Regulations 2004 is dependent on the following terms and conditions.

Sponsorship may be withdrawn if these Terms and Conditions are breached.

1. Commencement of the Research

In addition to Sponsor approval you must have the following in place before you can commence the study:

1.1. Favourable opinion from an appropriate NHS Research Ethics Committee (REC) and/or Health Research Authority before the trial can commence. A copy of the approval letter must be received by the Research Integrity and Governance (RIG) Team.

1.2. For a Clinical Trial of an Investigational Medicinal Product (CTIMP) MHRA approval in the form of a Clinical Trial Authorisation (CTA) must be acquired before the trial can commence. The investigator must register the trial on the European Clinical Trials Database to obtain a EudraCT number before applying for the CTA. A copy of the CTA must be received by the RIG Team.

1.3. For a clinical investigation for a medical device that is not CE marked for the purpose under investigation, MHRA approval in the form of a letter of 'no objection' must be acquired before the study can commence. A copy of the letter of 'no objection' must be received by the RIG team.

1.4. The Chief Investigator (CI) has ensured adequate funding is in place to conduct the study in full and in compliance with regulations.

1.5. A University Data Protection Impact Assessment (DPIA) has been completed.

1.6. The University Insurance Team are satisfied that all the necessary insurance and indemnities are in place.

1.7. The University Contracts Team are satisfied that all necessary contracts have been agreed and signed by all relevant parties.

1.8. Confirmation of capacity and capability from each participating NHS organisation (where required) and the necessary site agreements and/or Statement of Activities and Schedule of Events have been agreed.

1.9. Extension of a favourable ethical opinion (via ERGO and from NHS REC) will be required for any new site not included in the original ethics application before research can commence at that site.

1.10. The research must commence within 12 months of the date of approval of sponsorship. In the event that the research does not commence within 12 months the CI must provide the RIG Team and the relevant NHS REC with a written explanation for the delay.

1.11. In the event that the research does not commence within 24 months the UoS's agreement to act as Sponsor will be suspended and a new application for sponsorship must be submitted.

1.12. All researchers are responsible for checking whether they require a research passport with the relevant NHS Trust before commencing their research activity and where required to ensure this is in place before commencing.

1.13. For clinical trials, the CI must ensure that the study has been registered on a publicly accessible database such as www.clinicaltrials.gov before recruitment can begin and they must ensure that the information on the register is kept up to date.

1.14. For CTIMPs, device studies and challenge studies the Sponsor must do a final check that everything is in place and provide the 'green light' before commencing.

2. Conduct of the Research

2.1. All members of the research team must adhere to:

- The standards set out in the University's 'Code of Conduct for Research' and all other applicable University Policies which can be found on the University website (www.southampton.ac.uk/about/governance/regulations-policies-guidelines.page),
- All current applicable legislation and standards, including but not limited to, UK Policy Framework for Health and Social Care Research, International Conference on Harmonisation Good Clinical Practice Guidance (ICH-GCP), Human Tissue Act, Data Protection Act (2018),
- Medicines for Human Use (Clinical Trials) Regulations for CTIMPs,
- Medical Devices Directives,
- The Sponsor approved study protocol.

2.2. The CI must ensure that they and all members of the research team have the necessary expertise, experience and qualifications to fulfil the responsibilities delegated to them.

2.3. Any modifications, changes or amendments to the Sponsor approved protocol or changes in the conduct/management of a study must be submitted as an amendment through ERGO. The CI must wait for ERGO approval before submitting the change for approval to external bodies.

2.4. The CI must notify, and where necessary obtain approval from the relevant NHS REC, MHRA, HRA and NHS R&D Department before any modifications, changes or amendments to a research protocol or other change in the conduct/management of a study can be implemented.

2.5. The CI or a member of the study team must notify the RIG Team immediately if for any reason the CI is unable to continue in their role even if only temporarily.

3. Sponsor Responsibilities

The UoS as Sponsor will:

3.1. Provide the necessary authorisation for an IRAS submission to be made for HRA/NRES approval.

3.2. For CTIMPs provide the necessary authorisation for a CTA to be requested from the MHRA.

3.3. Check that all IMPs used in CTIMPs have the correct import licence in place (if applicable) and are supplied and/or manufactured in accordance with GMP or equivalent standards.

3.4. Ensure that all SUSAR reports are forwarded to the MHRA in accordance with applicable legislation and local policy and to have oversight of adverse events.

3.5. Ensure that the CI has evidenced the necessary expertise, experience and qualifications to conduct the study.

3.6. Provide a confirmation letter of approval of sponsorship.

3.7. Provide oversight of the monitoring activities for the research study.

3.8. Provide advice and guidance on study management, conduct and applicable legislation, guidelines and policies.

3.9. Audit and monitor studies as required to ensure the research is being conducted in compliance with applicable regulations, policies and guidelines.

3.10. Ensure the corrective actions and preventative actions are appropriate and actioned in the case of a GCP or protocol deviation or breach.

4. <u>Chief Investigator's Responsibilities</u>

The Chief Investigator must:

4.1 Ensure that they and all members of the research team will comply with University Policies and any regulations applicable to their research, including those set out by any professional bodies.

4.2 Ensure that the research team (including those external to UoS) only perform activities in accordance with the delegation log and the task allocation matrix.

4.3 Ensure that they and the research team complete the tasks allocated to them on the agreed task allocation matrix.

4.4 Obtain approval from the relevant NHS REC/HRA.

4.5 Obtain approval from the relevant NHS Trust R&D Department (where appropriate).

4.6 For CTIMPS, register the study on the European CT database, obtain a EudraCT number and obtain a CTA from the MHRA.

4.7 Ensure that all IMPs used in CTIMPs have the correct import licence in place (if applicable) and obtain the relevant documents to evidence that the IMP is supplied and/or manufactured in accordance with GMP.

4.8 Ensure appropriate storage and management of the IMP.

4.9 Maintain in a secure location a Trial Master File and where applicable an Investigator Site File. For CTIMPs, the TMF should contain all of the essential documents.

4.10 Allow members of the RIG team and other responsible members of UoS, access to members of the research team, relevant documents, equipment and devices for the purposes of monitoring (and if appropriate, where necessary, to patient records).

4.11 Record and review significant developments that may affect the study, particularly those that put the safety of the individuals at risk or affect the scientific value of the study, and report these developments to the RIG Team.

4.12 For CTIMPS, determine the seriousness, expectedness and causality of all adverse events and to ensure all SAEs and SUSARs are recorded and reported to the RIG team within 24 hours of becoming aware of them.

4.13 Prepare and submit an annual report as required by the NHS REC and provide a copy to the RIG team.

4.14 Maintain a record of all incidents that occur in the course of a research study and provide a copy of this report to the RIG team annually on the anniversary of our approval. This may be in the form of the NHS REC annual report or through the RIG Team self-monitoring form.

4.15 Request an extension for studies before the expected end date, if they are likely to overrun, and to notify any relevant bodies including the REC, MHRA and the funder if this is likely to happen. The extension of an end date does not require a submission of an amendment to NHS REC - it can be included in the annual report.

4.16 As required, inform the MHRA, the relevant NHS REC, the RIG team and where appropriate the NHS Trust of the end of the study within 90 days of its conclusion.

4.17 Inform the RIG team of any publications or Intellectual Property arising from the study.

4.18 Arrange for suitable archiving facilities, which must be organised prior to commencing the study. Such arrangements to take into account security and environmental considerations.

5. Termination of Sponsorship

Sponsorship may be suspended or terminated by UoS if:

5.1. One or more of the above conditions are not adhered to;

5.2. If there is poor or excessive recruitment, safety concerns or suspected fraud;

5.3. If any modifications, changes or amendments to the study result in the invalidation of one or more approvals;

5.4. If a CI leaves the study and an appropriate replacement is not found;

5.5. If a CI moves to a new place of employment but wish to continue to lead on the study; or

5.6. In the event of a Serious Breach in the conduct of the Study.
