# Supplementary material for: Treating Acute EXacerbations of COPD with Chinese HerbAL MedIcine to aid AntiBiotic Use Reduction (EXCALIBUR): study protocol of a randomised double-blind, placebo-controlled feasibility trial
Source: Pilot Feasibility Stud. 2022 Dec 19;8:262. doi: 10.1186/s40814-022-01224-8 (PMC9761047; doi:10.1186/s40814-022-01224-8)
Supplement: Supplementary file 5 — Appendix 5. PIS and consent form. [file 40814_2022_1224_MOESM5_ESM.docx]

Patient information sheet and Consent form
