# Supplementary figures and images for: Treating Acute EXacerbations of COPD with Chinese HerbAL MedIcine to aid AntiBiotic Use Reduction (EXCALIBUR): study protocol of a randomised double-blind, placebo-controlled feasibility trial
Source: Pilot Feasibility Stud. 2022 Dec 19;8:262. doi: 10.1186/s40814-022-01224-8 (PMC9761047; doi:10.1186/s40814-022-01224-8)

Appendix 6 Manufacturing Process and Process Control


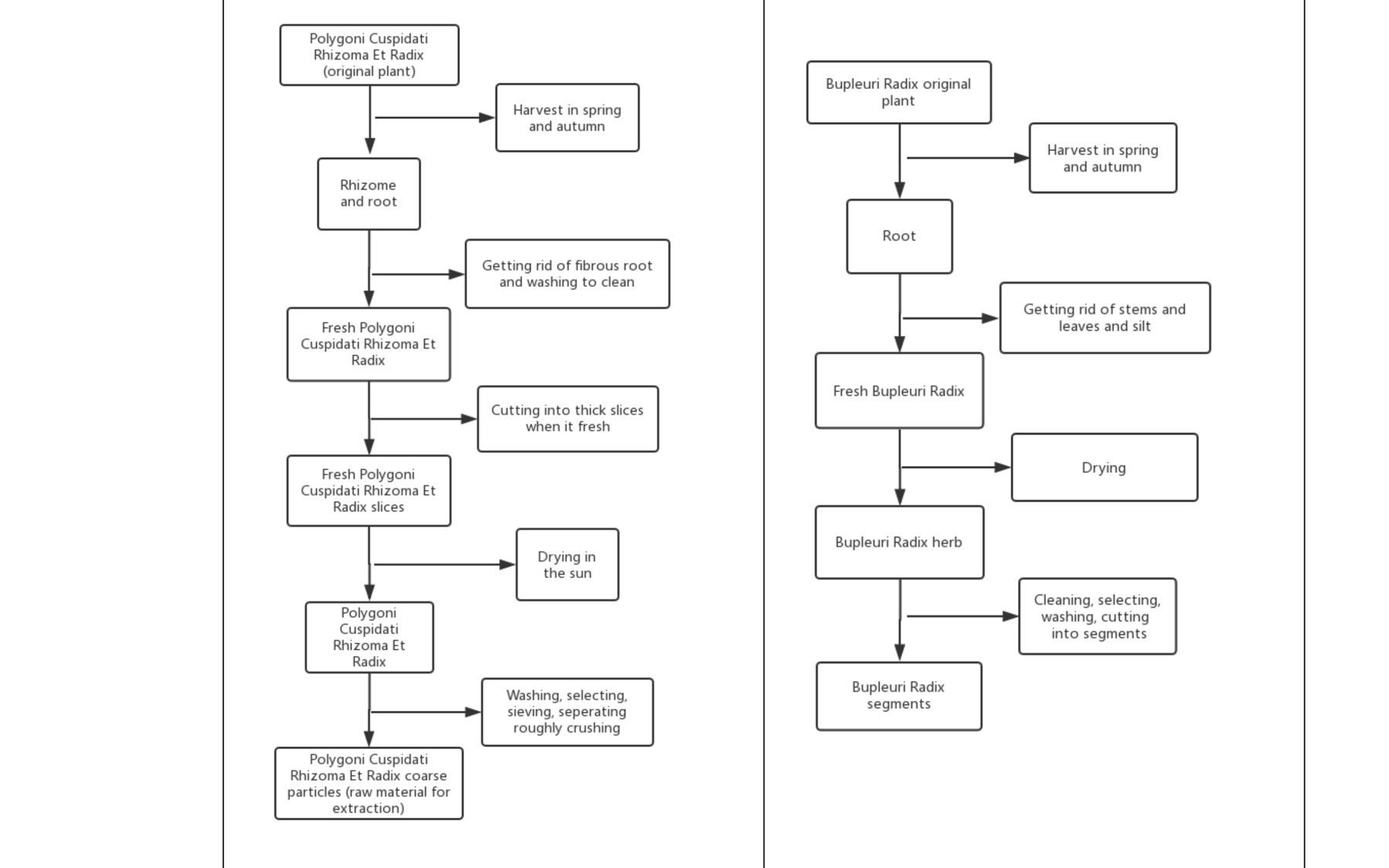


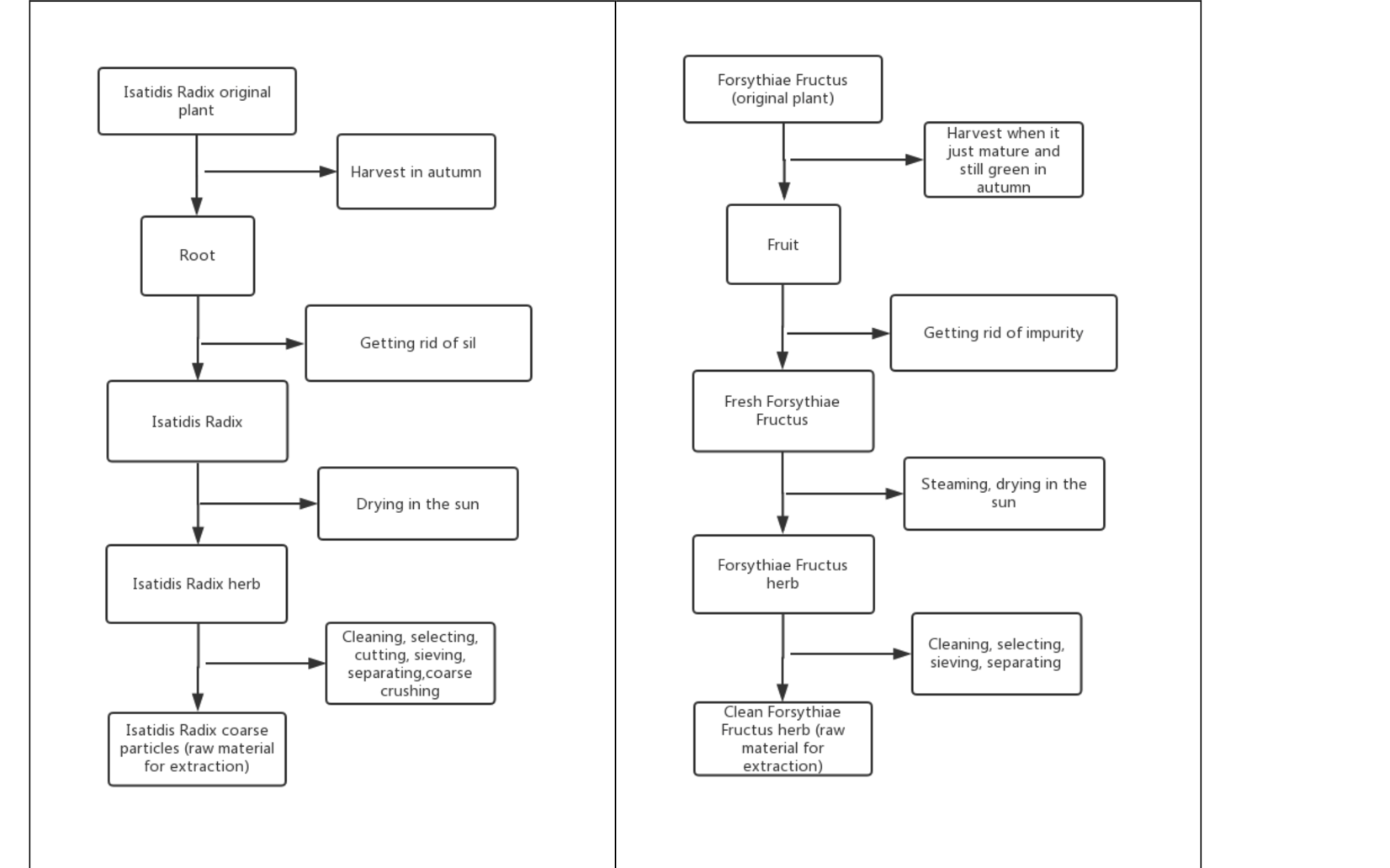


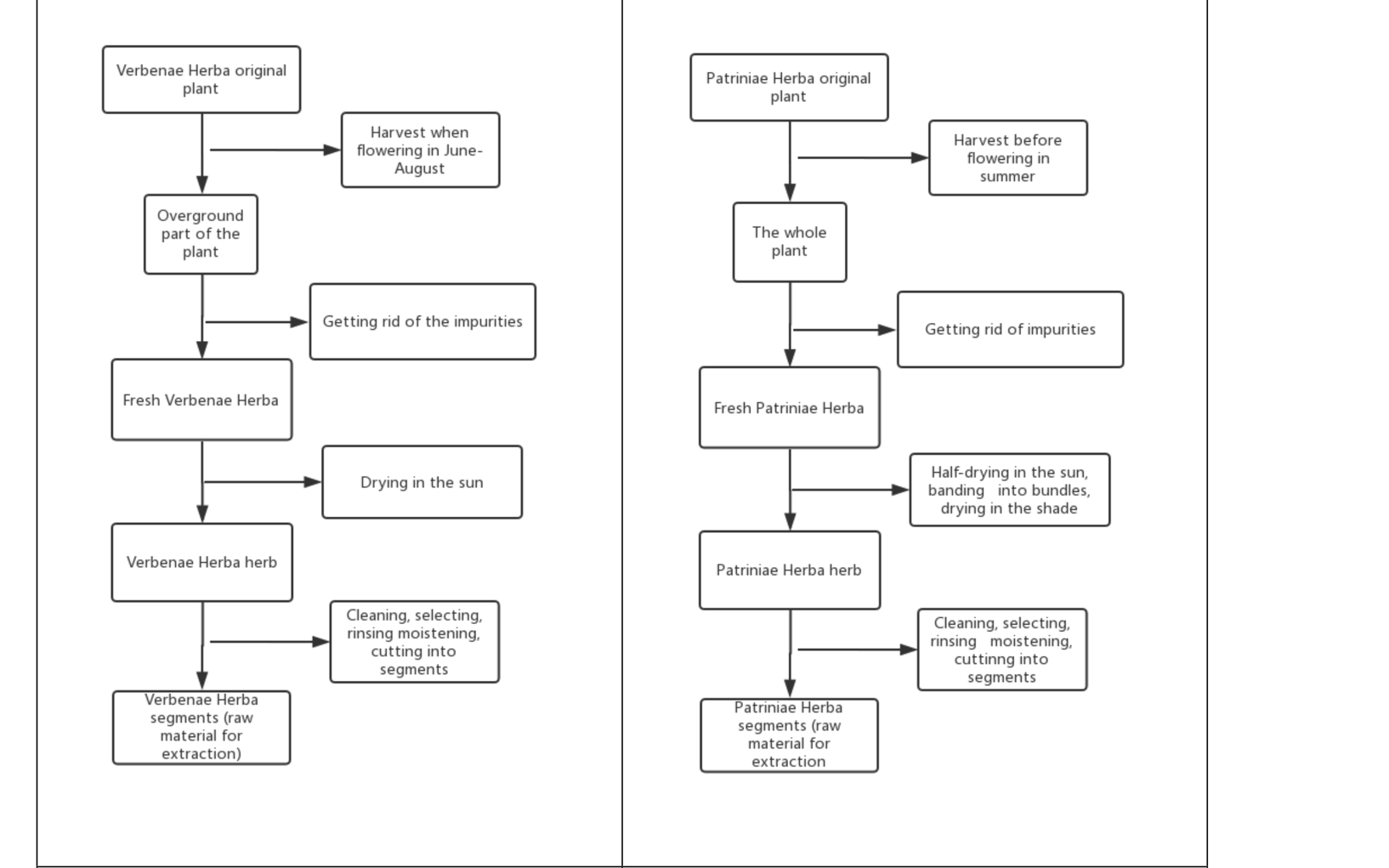


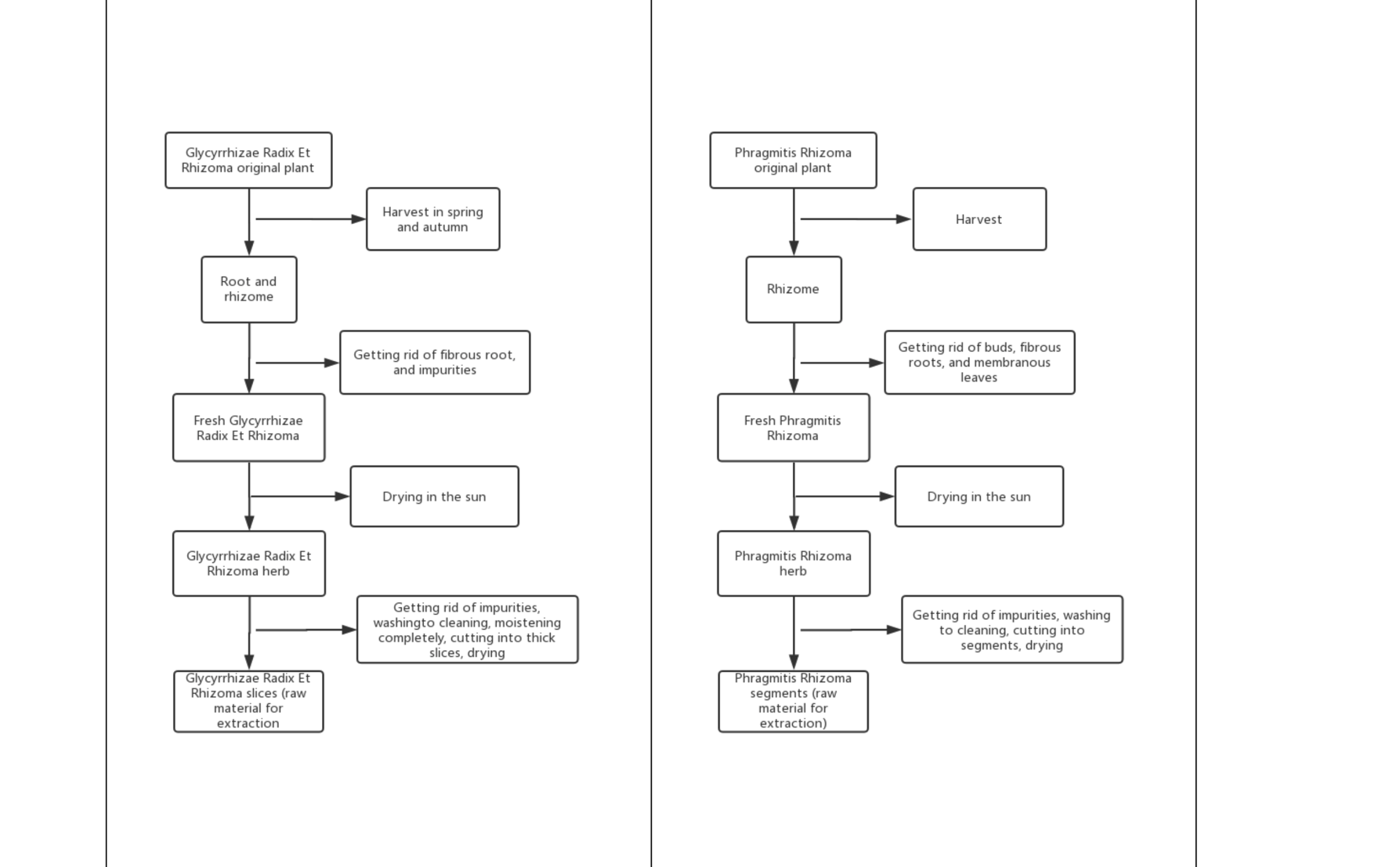

Supplement: Supplementary file 6 — Appendix 6. Flowcharts of the manufacturing process including cultivation, harvesting, drying and cleaning the herbs. [file 40814_2022_1224_MOESM6_ESM.docx]
