# Supplementary material for: Treating Acute EXacerbations of COPD with Chinese HerbAL MedIcine to aid AntiBiotic Use Reduction (EXCALIBUR): study protocol of a randomised double-blind, placebo-controlled feasibility trial
Source: Pilot Feasibility Stud. 2022 Dec 19;8:262. doi: 10.1186/s40814-022-01224-8 (PMC9761047; doi:10.1186/s40814-022-01224-8)
Supplement: Supplementary file 1 — Appendix 1. IRAS number 268737. [file 40814_2022_1224_MOESM1_ESM.pdf]

Ymchwil Iechyd a Gofal Cymru
Health and Care Research Wales

NHS
Health Research Authority

Professor Michael Moore
Professor of Primary Health Care
University of Southampton
Primary Care and Population Sciences
Primary Medical Care, Aldermoor Close
Southampton
SO16 5ST

Email: approvals@hra.nhs.uk
HCRW.approvals@wales.nhs.uk

30 November 2020

Dear Professor Moore

HRA and Health and Care Research Wales (HCRW) Approval Letter

**Study title:**	**Treating Acute EXacerbation of COPD with Chinese HerbAL MedIcine to aid AntiBiotic Use Reduction (EXCALIBUR) - Study of Shufeng JieduÂ® (SFJD) capsules to aid antibiotic use reduction in acute exacerbations of chronic obstructive pulmonary disease: a mixed-methods, double blind, randomised placebo-controlled feasibility trial**
**IRAS project ID:**	**268737**
**REC reference:**	**20/LO/0580**
**Sponsor**	**University of Southampton**

I am pleased to confirm that **HRA and Health and Care Research Wales (HCRW) Approval** has been given for the above referenced study, on the basis described in the application form, protocol, supporting documentation and any clarifications received. You should not expect to receive anything further relating to this application.

Please now work with participating NHS organisations to confirm capacity and capability, in line with the instructions provided in the "Information to support study set up" section towards the end of this letter.

How should I work with participating NHS/HSC organisations in Northern Ireland and Scotland?

HRA and HCRW Approval does not apply to NHS/HSC organisations within Northern Ireland and Scotland.

If you indicated in your IRAS form that you do have participating organisations in either of these devolved administrations, the final document set and the study wide governance report (including this letter) have been sent to the coordinating centre of each participating nation. The relevant national coordinating function/s will contact you as appropriate.

Please see IRAS Help for information on working with NHS/HSC organisations in Northern Ireland and Scotland.

How should I work with participating non-NHS organisations?
HRA and HCRW Approval does not apply to non-NHS organisations. You should work with your non-NHS organisations to obtain local agreement in accordance with their procedures.

What are my notification responsibilities during the study?

The standard conditions document "*After Ethical Review – guidance for sponsors and investigators*", issued with your REC favourable opinion, gives detailed guidance on reporting expectations for studies, including:

- Registration of research
- Notifying amendments
- Notifying the end of the study

The HRA website also provides guidance on these topics, and is updated in the light of changes in reporting expectations or procedures.

Who should I contact for further information?
Please do not hesitate to contact me for assistance with this application. My contact details are below.

Your IRAS project ID is **268737**. Please quote this on all correspondence.

Yours sincerely,
Alex Thorpe

Approvals Manager

Email: approvals@hra.nhs.uk

Copy to: *Dr Alison Knight, Sponsor's Representative*

List of Documents

The final document set assessed and approved by HRA and HCRW Approval is listed below.

Document	*Version*	*Date*
Confirmation of any other Regulatory Approvals (e.g. CAG) and all correspondence [EXCALIBUR MHRA confirmation of non-CTIMP]	1	20 June 2019
Contract/Study Agreement template [EXCALIBUR Template Site Agreement]	1	12 March 2020
Copies of advertisement materials for research participants [EXCALIBUR GP Poster]	1	10 February 2020
Covering letter on headed paper [Cover Letter EXCALIBUR]	1	24 March 2020
Covering letter on headed paper [Cover Letter EXCALIBUR]	1	06 November 2020
Evidence of Sponsor insurance or indemnity (non NHS Sponsors only) [EXCALIBUR UoS Sponsor Insurance Letter]	1	16 March 2020
GP/consultant information sheets or letters [EXCALIBUR Prompt Sheet for Clinicians]	1	10 February 2020
Interview schedules or topic guides for participants [EXCALIBUR-Q Recruited Interview Guide]	2	26 February 2020
Interview schedules or topic guides for participants [EXCALIBUR-Q Non-Recruited Interview Guide]	2	28 February 2020
IRAS Application Form [IRAS_Form_24032020]		24 March 2020
Letter from funder [EXCALIBUR Letter from Funder]	1	02 April 2019
Letter from sponsor [EXCALIBUR Sponsor Confirmation of Approval Letter]	1	16 March 2020
Letters of invitation to participant [EXCALIBUR Participant Invitation Letter for Mail Out]	1	10 February 2020
Letters of invitation to participant [EXCALIBUR Qualitative Invitation Letter for GP Practices]	1	07 February 2020
Non-validated questionnaire [EXCALIBUR Participant Diary]	1	10 February 2020
Organisation Information Document [EXCALIBUR Organisation Information Document]	2	05 June 2020
Other [EXCALIBUR SFJD Safety Dossier]	1	29 October 2020
Other [EXCALIBUR GP Safety Summary]	2	06 November 2020
Other [EXCALIBUR SFJD Patient Medication Information Leaflet]	2	06 November 2020
Other [EXCALIBUR Protocol Tracked Changes]	3	06 November 2020
Other [Response to Provisional Opinion - Ethical Review]	1	06 November 2020
Other [EXCALIBUR GCP for Chief Investigator]	1	10 September 2019
Other [EXCALIBUR Patient Contact Details Proforma]	1	10 March 2020
Other [EXCALIBUR Trial Risk Assessment]	1	28 February 2020
Participant consent form [EXCALIBUR Pregnancy Informed Consent Form]	1	25 February 2020
Participant consent form [EXCALIBUR Informed Consent Form]	3	06 November 2020
Participant consent form [EXCALIBUR Consent Form TC]	3	06 November 2020
Participant information sheet (PIS)	3	05 November 2020
Participant information sheet (PIS) [EXCALIBUR PIS TC]	3	05 November 2020
Participant information sheet (PIS) [EXCALIBUR Qualitative Substudy Patient Information Sheet]	2	04 November 2020
Participant information sheet (PIS) [EXCALIBUR Qualitative Substudy PIS TC]	2	04 November 2020
Participant information sheet (PIS) [EXCALIBUR Summary Patient	2	05 November 2020

Information Sheet for GP Practices]		
Participant information sheet (PIS) [EXCALIBUR Summary PIS for GP Practice]	2	05 November 2020
Participant information sheet (PIS) [EXCALIBUR Summary Patient Information Sheet for Mail Out]	2	05 November 2020
Participant information sheet (PIS) [EXCALIBUR Summary PIS for mail out]	2	05 November 2020
Research protocol or project proposal [EXCALIBUR - Protocol]	3	06 November 2020
Sample diary card/patient card [EXCALIBUR Participant Trial Card]	1	10 February 2020
Sample diary card/patient card [EXCALIBUR Patient Interest Card]	1	10 February 2020
Schedule of Events or SoECAT [EXCALIBUR Validated SoECAT]	1	12 March 2020
Summary CV for Chief Investigator (CI) [EXCALIBUR CV for Chief Investigator]	1	16 February 2017
Summary CV for Chief Investigator (CI) [Michael Moore CI - CV]	1	24 March 2020
Validated questionnaire [EXCALIBUR Final CAT Questionnaire]	1	10 February 2020
Validated questionnaire [EXACT-PRO Questionnaire]	1.1	01 January 2013

IRAS project ID	268737

Information to support study set up

The below provides all parties with information to support the arranging and confirming of capacity and capability with participating NHS organisations in England and Wales. This is intended to be an accurate reflection of the study at the time of issue of this letter.

Types of participating NHS organisation	Expectations related to confirmation of capacity and capability	Agreement to be used	Funding arrangements	Oversight expectations	HR Good Practice Resource Pack expectations
All sites will perform the same research activities therefore there is only one site type.	Research activities should not commence at participating NHS organisations in England or Wales prior to their formal confirmation of capacity and capability to deliver the study.	The sponsor is using a bespoke agreement. The HRA and HCRW take no position on the acceptability of these changes. Participating NHS organisations should now determine its acceptability and liaise with the sponsor to confirm the content of the agreement.	External study funding has been sought.	A Principal Investigator should be appointed at study sites.	No Honorary Research Contracts, Letters of Access or pre-engagement checks are expected for local staff employed by the participating NHS organisations. Where arrangements are not already in place, network staff (or similar) undertaking any of the research activities listed in the IRAS form (except for administration of questionnaires or surveys), would be expected to obtain an honorary research contract from one NHS organisation (if university employed), followed by Letters of Access for subsequent organisations. This would be on the basis of a Research Passport (if university employed) or an NHS to NHS confirmation of pre-engagement checks letter (if NHS employed). These should confirm enhanced DBS checks, including appropriate barred list checks, and occupational health clearance. For research team members only administering questionnaires or surveys, a Letter of Access based on enhanced DBS checks and occupational health clearance would be appropriate.

Other information to aid study set-up and delivery

This details any other information that may be helpful to sponsors and participating NHS organisations in England and Wales in study set-up.
The applicant has indicated that they intend to apply for inclusion on the NIHR CRN Portfolio.
