# Supplementary material for: Treating Acute EXacerbations of COPD with Chinese HerbAL MedIcine to aid AntiBiotic Use Reduction (EXCALIBUR): study protocol of a randomised double-blind, placebo-controlled feasibility trial
Source: Pilot Feasibility Stud. 2022 Dec 19;8:262. doi: 10.1186/s40814-022-01224-8 (PMC9761047; doi:10.1186/s40814-022-01224-8)
Supplement: Supplementary file 3 — Appendix 3. Ethical approval: REC reference 20/LO/0580. [file 40814_2022_1224_MOESM3_ESM.pdf]

NHS
Health Research Authority

London - Surrey Research Ethics Committee
Nottingham Centre
The Old Chapel
Royal Standard Place
Nottingham
NG1 6FS

<u>Please note</u>: This is the favourable opinion of the REC only and does not allow you to start your study at NHS sites in England until you receive HRA Approval

30 November 2020

Professor Michael Moore
Professor of Primary Health Care
University of Southampton
Primary Care and Population Sciences
Primary Medical Care, Aldermoor Close
Southampton
SO16 5ST

Dear Professor Moore

**Study title:**	**Treating Acute EXacerbation of COPD with Chinese HerbAL MedIcine to aid AntiBiotic Use Reduction (EXCALIBUR) - Study of Shufeng Jiedu® (SFJD) capsules to aid antibiotic use reduction in acute exacerbations of chronic obstructive pulmonary disease: a mixed-methods, double blind, randomised placebo-controlled feasibility trial**
**REC reference:**	**20/LO/0580**
**IRAS project ID:**	**268737**

Thank you for your letter of 06 November 2020, responding to the Committee's request for further information on the above research and submitting revised documentation.

The further information has been considered on behalf of the Committee by the Chair.

Confirmation of ethical opinion

On behalf of the Committee, I am pleased to confirm a favourable ethical opinion for the above research on the basis described in the application form, protocol and supporting documentation as revised, subject to the conditions specified below.

Conditions of the favourable opinion

The REC favourable opinion is subject to the following conditions being met prior to the start of the study.

Confirmation of Capacity and Capability (in England, Northern Ireland and Wales) or NHS management permission (in Scotland) should be sought from all NHS organisations involved in the study in accordance with NHS research governance arrangements. Each NHS organisation must confirm through the signing of agreements and/or other documents that it has given permission for the research to proceed (except where explicitly specified otherwise).

Guidance on applying for HRA and HCRW Approval (England and Wales)/ NHS permission for research is available in the Integrated Research Application System.

For non-NHS sites, site management permission should be obtained in accordance with the procedures of the relevant host organisation.

Sponsors are not required to notify the Committee of management permissions from host organisations

Registration of Clinical Trials

It is a condition of the REC favourable opinion that **all clinical trials are registered** on a publicly accessible database. For this purpose, 'clinical trials' are defined as the first four project categories in IRAS project filter question 2. Registration is a legal requirement for clinical trials of investigational medicinal products (CTIMPs), except for phase I trials in healthy volunteers (these must still register as a condition of the REC favourable opinion).

Registration should take place as early as possible and within six weeks of recruiting the first research participant at the latest. Failure to register is a breach of these approval conditions, unless a deferral has been agreed by or on behalf of the Research Ethics Committee (see here for more information on requesting a deferral: https://www.hra.nhs.uk/planning-and-improving-research/research-planning/research-registration-research-project-identifiers/

As set out in the UK Policy Framework, research sponsors are responsible for making information about research publicly available before it starts e.g. by registering the research project on a publicly accessible register. Further guidance on registration is available at: https://www.hra.nhs.uk/planning-and-improving-research/research-planning/transparency-responsibilities/

You should notify the REC of the registration details. We will audit these as part of the annual progress reporting process.

It is the responsibility of the sponsor to ensure that all the conditions are complied with before the start of the study or its initiation at a particular site (as applicable).

After ethical review: Reporting requirements

The attached document "After ethical review – guidance for researchers" gives detailed guidance on reporting requirements for studies with a favourable opinion, including:

- Notifying substantial amendments
- Adding new sites and investigators
- Notification of serious breaches of the protocol
- Progress and safety reports
- Notifying the end of the study, including early termination of the study
- Final report

The latest guidance on these topics can be found at https://www.hra.nhs.uk/approvals-amendments/managing-your-approval/.

Ethical review of research sites

NHS/HSC sites

The favourable opinion applies to all NHS/HSC sites listed in the application subject to confirmation of Capacity and Capability (in England, Northern Ireland and Wales) or management permission (in Scotland) being obtained from the NHS/HSC R&D office prior to the start of the study (see "Conditions of the favourable opinion" below).

Non-NHS/HSC sites

I am pleased to confirm that the favourable opinion applies to any non-NHS/HSC sites listed in the application, subject to site management permission being obtained prior to the start of the study at the site.

Approved documents

The final list of documents reviewed and approved by the Committee is as follows:

Document	*Version*	*Date*
Confirmation of any other Regulatory Approvals (e.g. CAG) and all correspondence [EXCALIBUR MHRA confirmation of non-CTIMP]	1	20 June 2019
Copies of advertisement materials for research participants [EXCALIBUR GP Poster]	1	10 February 2020
Covering letter on headed paper [Cover Letter EXCALIBUR]	1	06 November 2020
Evidence of Sponsor insurance or indemnity (non NHS Sponsors only) [EXCALIBUR UoS Sponsor Insurance Letter]	1	16 March 2020
GP/consultant information sheets or letters [EXCALIBUR Prompt Sheet for Clinicians]	1	10 February 2020
Interview schedules or topic guides for participants [EXCALIBUR-Q Recruited Interview Guide]	2	26 February 2020
Interview schedules or topic guides for participants [EXCALIBUR-Q Non-Recruited Interview Guide]	2	28 February 2020

IRAS Application Form [IRAS_Form_24032020]		24 March 2020
Letter from funder [EXCALIBUR Letter from Funder]	1	02 April 2019
Letter from sponsor [EXCALIBUR Sponsor Confirmation of Approval Letter]	1	16 March 2020
Letters of invitation to participant [EXCALIBUR Participant Invitation Letter for Mail Out]	1	10 February 2020
Letters of invitation to participant [EXCALIBUR Qualitative Invitation Letter for GP Practices]	1	07 February 2020
Non-validated questionnaire [EXCALIBUR Participant Diary]	1	10 February 2020
Other [EXCALIBUR GCP for Chief Investigator]	1	10 September 2019
Other [EXCALIBUR Patient Contact Details Proforma]	1	10 March 2020
Other [EXCALIBUR Trial Risk Assessment]	1	28 February 2020
Other [EXCALIBUR SFJD Safety Dossier]	1	29 October 2020
Other [EXCALIBUR GP Safety Summary]	2	06 November 2020
Other [EXCALIBUR SFJD Patient Medication Information Leaflet]	2	06 November 2020
Other [EXCALIBUR Protocol Tracked Changes]	3	06 November 2020
Other [Response to Provisional Opinion - Ethical Review]	1	06 November 2020
Participant consent form [EXCALIBUR Pregnancy Informed Consent Form]	1	25 February 2020
Participant consent form [EXCALIBUR Informed Consent Form]	3	06 November 2020
Participant consent form [EXCALIBUR Consent Form TC]	3	06 November 2020
Participant information sheet (PIS) [EXCALIBUR Qualitative Substudy Patient Information Sheet]	1	10 February 2020
Participant information sheet (PIS) [EXCALIBUR Pregnancy Patient Information Sheet]	1	25 February 2020
Participant information sheet (PIS) [EXCALIBUR Summary Patient Information Sheet for GP Practices]	1	10 February 2020
Participant information sheet (PIS) [EXCALIBUR Summary Patient Information Sheet for Mail Out]	1	10 February 2020
Participant information sheet (PIS)	3	05 November 2020
Participant information sheet (PIS) [EXCALIBUR PIS TC]	3	05 November 2020
Participant information sheet (PIS) [EXCALIBUR Qualitative Substudy Patient Information Sheet]	2	04 November 2020
Participant information sheet (PIS) [EXCALIBUR Qualitative Substudy PIS TC]	2	04 November 2020
Participant information sheet (PIS) [EXCALIBUR Summary Patient Information Sheet for GP Practices]	2	05 November 2020
Participant information sheet (PIS) [EXCALIBUR Summary PIS for GP Practice]	2	05 November 2020
Participant information sheet (PIS) [EXCALIBUR Summary Patient Information Sheet for Mail Out]	2	05 November 2020
Participant information sheet (PIS) [EXCALIBUR Summary PIS for mail out]	2	05 November 2020
Research protocol or project proposal [EXCALIBUR - Protocol]	3	06 November 2020
Sample diary card/patient card [EXCALIBUR Participant Trial Card]	1	10 February 2020
Sample diary card/patient card [EXCALIBUR Patient Interest Card]	1	10 February 2020
Summary CV for Chief Investigator (CI) [EXCALIBUR CV for Chief Investigator]	1	16 February 2017

Summary CV for Chief Investigator (CI) [Michael Moore CI - CV]	1	24 March 2020
Validated questionnaire [EXCALIBUR Final CAT Questionnaire]	1	10 February 2020
Validated questionnaire [EXACT-PRO Questionnaire]	1.1	01 January 2013

Statement of compliance

The Committee is constituted in accordance with the Governance Arrangements for Research Ethics Committees and complies fully with the Standard Operating Procedures for Research Ethics Committees in the UK.

User Feedback

The Health Research Authority is continually striving to provide a high quality service to all applicants and sponsors. You are invited to give your view of the service you have received and the application procedure. If you wish to make your views known please use the feedback form available on the HRA website:
http://www.hra.nhs.uk/about-the-hra/governance/quality-assurance/

HRA Learning

We are pleased to welcome researchers and research staff to our HRA Learning Events and online learning opportunities– see details at:
https://www.hra.nhs.uk/planning-and-improving-research/learning/

IRAS project ID: 268737 Please quote this number on all correspondence

With the Committee's best wishes for the success of this project.

Yours sincerely

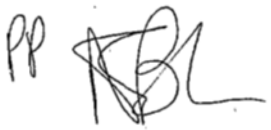

Mrs Chrissie Lawson
Chair

Email: surrey.rec@hra.nhs.uk

Enclosures: "After ethical review – guidance for researchers"

Copy to: Dr Alison Knight
